# Minutes of the Georgia Medical Association

**Published:** 1873-04

**Authors:** 


### MINUTES OF THE GEORGIA MEDICAL ASSOCIATION.

### FIRST DAY—MORNING SESSION.

ATLANTA, April 9th, 1873.

The Medical Association of Georgia convened this day, in the Senate Chamber, in its twenty-fourth annual session, and was called to order by the President, Dr. G. W. Holmes, of Rome.

Prayer was offered by Rev. Dr. Wills.

Dr. J. F. Alexander, on behalf of the Committee of Arrangements, and the physicians of Atlanta, welcomed the Association to the city. He was followed by Gen. A. C. Garlington in behalf of the citizens. Dr. R. T. Kendrick, of Calhoun county, by request of the President, returned the thanks of the Association for the kind wishes and attentions.

The Chairman of the Committee of Arrangements requested that the time be extended till the afternoon for the report on the credentials of members.

Also announced the following programme for the entertainment of the members during their stay in the city:

*First Day, Wednesday*—Evening receptions complimentary to the Association, by Gov. and Mrs. James M. Smith, at the Executive Mansion on Peachtree street; Mr. and Mrs. A. Leyden, at their residence on Peachtree street; Dr. and Mrs. W. F. Westmoreland, Marietta street.

*Second Day, Thursday*—At 12 o'clock M., the annual oration will be delivered by the orator, Dr. J. T. Johnson.

Immediately after adjournment of the morning session, by invitation of Mr. Mallon, Superintendent of the Public Schools, the Association will visit several of the schools of the city, and witness their operations, and the calisthenic exercises by the girls of the High School.

Thursday evening, at 8 o'clock, an entertainment will take place at the Kimball House, entrance on Decatur street.

*Third Day, Friday*—At 1 o'clock of the afternoon, the members and their friends will take the street cars on Marietta street, at the door of the capitol, and proceed, by invitation of Mr. Schofield, President of the Company, to the Schofield Rolling Mill, where they will have the opportunity of witnessing this mammoth institution in full blast, and be returned to the city by the usual dinner hour.

On Friday afternoon, a complimentary drive will be given to the members of the Association; starting from the capital at 4 o'clock, they will visit the famous Ponce DeLeon and Healing Springs, and other interesting points in and around the city.

The President then delivered the annual address.

Dr. W. A. Love, of Atlanta, introduced the following, which was adopted:

*Resolved*, That the annual address of the President be referred to a special committee, consisting of Drs. J. P. Logan, E. J. Kirkscey and DeSaussure Ford, for consideration and report upon its suggestion.

The Association adjourned to meet at 2½ o'clock, P.M.

AFTERNOON SESSION.

Association called to order by the President.

Dr. A. W. Griggs, chairman of the committee on the Constitution, read the new Constitution sumbitted by that committee.

By resolution of Dr. DeSaussure Ford, the Secretary read the Constitution then in force.

The new Constitution was then read and adopted by sections. The sections were all adopted without objection, except those relating to the authority of the censors and the time of holding the annual meetings. These, after full discussion, received several amendments. The annual meeting is hereafter to occur on the first Wednesday in April, instead of the second Wednesday as heretofore; the name of the body is changed to "The Medical Association of Georgia"—formerly the "Georgia Medical Association."

The Constitution was then adopted as a whole. (Will be published in full in the forthcoming volume of Transactions.)

Dr. E. J. Kirkscey introduced resolutions, which were adopted, inviting the clergy, city officials, and members of the press, to seats on the floor; also surgeons of the army and members of the profession of other States, and that they register their names and address; also, "that the addresses of Dr. J. F. Alexander and Gen. A. C. Garlington be secured and referred to the Committee on Publication."

Dr. Robert Battey introduced a resolution that the President appoint a committee of five to nominate suitable candidates for the Board of Censors. Adopted: and Drs. Battey, Alexander, Musgrove, Ford and Griggs were appointed.

The resignation of Dr. S. H. Stout, permanent Secretary, induced by conflicting business engagements, was accepted by the Association. A vote of thanks was passed for the kind wishes he expressed, and for the faithful manner in which he had served the Association.

The Secretary read the resignation of membership of Dr. W. T. Grant, of Brunswick; also of Dr. C. L. Redwine, of Atlanta. Referred to the Board of Censors.

The committee for nominating a Board of Censors, reported:

For five years—Robert Battey, M.D., of Rome.

For four years—W. H Doughty, M.D., Augusta.

For three years—C. B. Nottingham, M.D., Macon.

For two years—J. F. Alexander, M.D., Atlanta.

For one year—J. G. Thomas, M.D., Savannah.

These were elected by the adoption of the Committee's report.

There not being a quorum of the Censors present, the President appointed Dr. DeSaussure Ford, of Augusta, to act as censor during the session.

Dr. H. H. Smith, of Scriven, chairman of the committee for memorializing the Legislature of the State in reference to a physician's lien, reported, by letter, that he had visited the capital in the interest of the measure, but arrived too late in the session to induce that body to consider any new matter. At his request, the committee was continued.

Dr. Battey, delegate to the Alabama Medical Association, presented his report. Referred to Committee on Publication.

The committee for petitioning the Legislature relative to births, marriages and deaths, reported that in the present state of public sentiment, they had thought it useless or inexpedient to present the matter to the Legislature; that much labor, through the press and other channels, would be requisite for educating the sentiment of the people and the General Assembly to an appreciation of its importance. Report received.

The Board of Censors submitted the names of the following persons as suitable candidates for membership: J. W. Lambert. Grantville; J. A. Dozier, Wrightsboro; R. Warren, Dalton; J. C. Bivings, Dalton; Jno. T. Dixon, Woodbury; Wm. B. Wells, Red Clay; J. J. Pursell, Hamilton; E. D. Alfriend, Sparta; Thos. S. Hopkins, Thomasville; A. K. Smith, U. S. A., Atlanta; Paul Faver, Fayetteville; W. L. Williams, Fayetteville; C. M.

Hill, Atlanta; E. N. Branham, Covington; F. L. Wisdom, Buena Vista; D. DuPre, Atlanta; J. C. Cook, Columbus; J. W. McFaul, Atlanta; H. B. Lee, Atlanta.

These were elected members of the Association by the unanimous adoption of the report of the censors.

Dr. Battey read the report of Dr. W. D. Hoyt, of Rome, chairman of the Committee on Inebriate Asylums. Report adopted.

A motion was adopted that the President appoint a committee to confer with a committee selected by the Legislature for the consideration of the subject in question.

Dr. W. D. Hoyt, chairman of the Committee for corresponding with members charged with a violation of the Code of Ethics, submitted, through Dr. Battey, a report which was referred, with papers, to the Board of Censors.

Adjourned to meet at 8½ o'clock on Thursday morning.

---

SECOND DAY—MORNING SESSION.

The Association was called to order at 9 o'clock, by the President.

Dr. W. F. Westmoreland moved to reconsider that section of the Constitution fixing the time of the annual meeting on the first Wednesday in April.

The President ruled the motion out of order, as the question had been reconsidered and laid on the table on the previous day.

On motion, the President appointed Drs. Kirkscey, McDowell and Wells a committee to audit the accounts of the Treasurer. The committee soon made report that the accounts were correct, and a balance of twenty cents in the treasury.

Dr. Robert Battey, of Rome, read a paper on "Normal Ovariotomy." This, on motion, was referred to a special committee of five to be appointed by the President. Drs. W. F. Westmoreland, G. M. McDowell, W. C. Musgrove, J. F. Bozeman and C. D. Smith, committee.

The Board of Censors, through Dr. Battey, reported favorably on the application for membership of the following persons: Dr. T. W. Sims, Covington; Dr. J. S. Simmons, Gainesville; Dr. J. P. Stevens, Wooten, Lee county; Dr. W. G. Drake, Atlanta; Dr. S. W. Palmer, Atlanta; Dr. Jas. A. Long, LaGrange. Also, that the names of T. S. Tuggle, of Columbus, and F. O. Dannelly, be dropped from the roll of membership. The report was adopted.

Dr. D. W. Hammond, of Macon, chairman of Committee on Surgery for the Fourth Congressional District, presented, by proxy, papers which were read and referred to the Committee on Publication. These papers are: one by Dr. D. W. Hammond, "Cutting Throat, Operation Unsuccessful—Performed by the Patient;" one by Dr. C. B. Nottingham, of Macon, "Ovariotomy—Death;" and one by Dr. G. E. Sussdorf, of Macon, "Tracheotomy."

Dr. W. O'Daniel, of Twiggs county, read the following papers: "Depression of the Cranium;" "Tracheotomy;" "Placenta Prævia;" "Strangulated Hernia;" "Phthisis Pulmonalis;" "Wound of Right Lung;" "Wound of Head and Brain." These were referred to the Committee on Publication.

At 12 o'clock the annual address was delivered by the orator, Dr. J. T. Johnson, of Atlanta. A resolution of thanks was tendered.

The Association then adjourned to visit the Public Schools of the city, and to convene again at 2½ o'clock.

### SECOND DAY—AFTERNOON SESSION.

The Association was called to order by Dr. A. W. Griggs, second Vice-President.

Dr. E. J. Kirkscey presented two papers by Dr. Carlisle Terry, of Columbus—"A Thesis on Climatology," and a "Report on Surgery." These were referred to the Committee on Publication.

Dr. DeSaussure Ford read some papers for Dr. W. H. Doughty, of Augusta. Referred to Committee on Publication. (The titles not remembered, the papers being in the hands of the author for completion or amendment.)

The following report was presented by Dr. J. P. Logan:

"The special committee to whom was referred the President's address, beg leave to make the following report: It is in our judgment an eminently judicious and practical address, and we recommend that it be published in the Transactions.

"In regard to its special recommendations, the substance of one having been already adopted by this body, it is unnecessary for us to make further comment upon it.

"In regard to the other, providing for a State Board of Health, we are strongly impressed with its importance, and recommend its adoption by this body, in the following resolution:

"*Resolved*, That a committee of five of the members of this body shall be appointed by the President, a majority of whom shall reside at the seat of government, to memorialize the Legislature for the establishment of a State Board of Health."

Report adopted.

Dr. E. J. Kirkscey moved an assessment of three (3) dollars upon each member. This was amended by the insertion on five (5) instead of three (3) dollars, and carried.

Dr. E. D. Alfriend, of Sparta, moved that should this amount not prove sufficient for the expenses of the Association, the absent members be called on for their dues.

Dr. Kirkscey presented his report as delegate from this body to the Arkansas Medical Association. Report received and adopted.

Dr. Ford presented a specimen of tumor of the bones composing the knee, with an account of the operations for its relief. The paper not being fully completed, he was requested to submit it to the Committee on Publication after he should prepare it.

The resignation of Dr. J. Stainback Wilson, of Atlanta, was read and referred to the Board of Censors.

Dr. J. P. Logan presented a hydatid tumor from the uterus.

The Secretary proceeded with the call for report of the sections of the Congressional Districts.

Dr. K. P. Moore, of Knoxville, Crawford county, from the Section of Practice of Third District, read a paper on the muriate of ammonia. Referred to the Committee on Publication.

The Board of Censors reported favorably on the application for membership of Drs. Wm. J. Johnson, of Atlanta, and T. R. Kendall, of Thomaston, Upson county; also, that the resignation of Dr. J. Stainback Wilson be accepted. Report adopted.

Dr. V. H. Taliaferro, of Atlanta, Chairman of Committee on Gynæcology of Third District, (having removed his residence since appointed) presented specimen of a uterine tumor, and of enlarged uterus, with remarks. Requested to write out the case cases and submit them to the Committee on Publication.

The report of Dr. D. W. Hammond, chairman of the Section of Surgery for the Fourth District, had been previously read, out of the regular order.

Dr. Logan stated that Dr. R. D. Moore, of Athens, chairman of the Section on Practice of Medicine for the Sixth District, had intended offering a report, but sickness in his family had prevented its completion. Was requested to complete it, and deliver it to the Committee on Publication.

Dr. W. S. Armstrong, of Atlanta, read a paper " Œdema of Scrotum." Referred to Committee on Publication.

Dr. J. G. Westmoreland read a paper on " Cerebro-Spinal Meningitis," advocating its acute inflammatory nature and corresponding treatment. This was briefly discussed by Drs. White, Rauschenberg and Johnson. Paper received and referred.

The Board of Censors reported, recommending that the resignation of Drs. C. L. Redwine and W. T. Grant be accepted; also, as candidate for membership, Dr. W. S. Zellars, Palmetto, Campbell county. Report adopted.

Dr. Kirkscey presented a paper for Dr. T. J. Word, of Columbus. Referred to Committee on Publication.

Dr. A. W. Griggs presented a report on "Pulmonary Tuberculosis." Referred to Committee on Publication.

Dr. Kirkscey presented some samples of fluid meat extract, desiring the members to test it. It was referred, by motion, to Dr. Taliaferro for opinion.

The Board of Censors reported the name of Dr. J. C. C. Blackburn, of Barnesville, Pike county, as applicant for membership. Report adopted.

The Association adjourned to meet at 8½ o'clock the next morning.

---

THIRD DAY—MORNING SESSION.

The Association was called to order by the President.

The special order for the morning, the discussion of cerebro-spinal meningitis, was postponed for the present.

The regular order was suspended, and Dr. Word's paper, which was referred yesterday by title, was taken up and read by Dr. Battey.

Dr. W. F. Westmoreland, chairman Section on Surgery for the Seventh District, presented some specimens of osteo-sarcoma and cystic tumor of jaw. He was requested to prepare the papers and submit them to the Committee on Publication.

Dr. D. G. Hunt, of Dalton, presented a paper, through Dr. Battey, giving the operation of trephining for the removal of a knife-blade imbedded in the skull and penetrating the brain. The blade and the button of bone removed were exhibited. The patient's recovery was perfect.

Dr. V. H. Taliaferro made some remarks discussing the question of the correctness of diagnosis by the reported case of cure of ovarian tumor, by the muriate of ammonia, by Dr. Word.

Dr. K. P. Moore followed, confirming Dr. Taliaferro in the opinion as to the difficulty of diagnosis in such cases.

Dr. Ch. Rauschenburg, of Atlanta, read a paper, "Origin, Nature and Treatment of Fever, in the Light of Modern Physiolology." Referred to Committee on Publication.

The Board of Censors reported the name of Dr. D. S. Brandon, Thomasville, as a suitable candidate for membership. Elected by adoption of the report.

The President announced the committee for nominating the officers, consisting of one member from each county represented. Committee instructed to prepare their report.

The President appointed the following members as delegates to the meeting of the American Medical Association : Drs. J. P. Logan, Atlanta ; DeSaussure Ford, Augusta ; G. M. McDowell, Barnesville ; W. F. Westmoreland, Atlanta ; W. H. Doughty, Augusta ; R. D. Moore, Athens ; T. J. Word, Columbus ; C. B. Nottingham, Macon ; Robert Battey, Rome ; F. A. Stanford, Columbus ; J. G. Thomas, Savannah ; Juriah Harriss, Savannah; C. D. Smith, Newnan ; Paul Faver, Fayetteville ; D. T. Johnson, Griffin ; J. T. Banks, Griffin ; K. P. Moore, Knoxville ; W. S. Armstrong, Atlanta ; E. J. Kirkscey, Columbus ; T. J. Brewster, Columbus ; J. T. Slaughter, Carroll county ; W. A. Culberson, Cave Spring ; D. W. Hammond, Macon ; G. J. Grimes, Columbus ; A. B. Calhoun, Newnan ; J. F. Alexander, Atlanta ; R. P. Myers, Savannah ; A. G. Whitehead, Burke county.

On motion of Dr. Kirkscey, the President appointed a committee of three to arrange the Sections with reference to the recent remodeling of the Congressional Districts. Committee—Drs. E. J. Kirkscey, H. V. M. Miller and Robert Battey.

The President announced the Committee on Inebriate Asylum, to confer with a like committee of the State Legislature, Drs. W. D. Hoyt, G. M. McDowell and J. P. Logan.

The Board of Censors reported favorably upon the application for membership of Drs. T. D. Longino, Palmetto, Campbell county; W. L. Williams; M. W. Gray, Cave Spring; E. P. Taylor, Thomasville. These were elected by adoption of the report.

Dr. C. B. Nottingham read a bill he had prepared to present to the Legislature of the State, for the registration of births, deaths and marriages. He then offered the following:

*Resolved,* That the paper in the form of a bill in regard to births, deaths, etc., be referred to a committee of seven, to be presented to the next Legislature, with an appropriate memorial urging its enactment into law.

2. *Resolved,* That should the committee be successful in securing the passage of the measure through the Legislature, they stand instructed to furnish the Comptroller the forms, nomenclature, etc., that may be requisite to enable his officials in the several counties to so make their returns as to be interesting and serviceable to this Association.

The chair appointed on the committee, Drs. Nottingham, W. F. Westmoreland, Battey, Goldsmith, Miller, Griggs and Cooper.

Dr. G. G. Crawford, of Atlanta, exhibited to the Association a patient from whom he had removed, during the late war, four inches of the shaft of the humerus. Leaving the periosteum intact, the formation of new bone was sufficient to allow almost perfect function of the arm. Dr. Crawford was requested to furnish a history of the case for publication.

Dr. J. P. Logan, chairman of the Nominating Committee, reported the following candidates for officers for the ensuing year:

President—Dr. W. F. Westmoreland, Fulton county.

1st Vice-President—Dr. DeSaussure Ford, Richmond county.

2d Vice-President—Dr. Samuel G. White, Baldwin county.

Secretary—Dr. J. T. Johnson, Fulton county.

Treasurer—Dr. W. O'Daniel, Twiggs county.

These officers were elected by the adoption of the Committee's report.

[The President and Vice-Presidents are elected for one year, the Secretary and Treasurer for five years.]

The new Constitution empowers the President to appoint the orator. The President appointed to that position, Dr. R. B. Ridley, of LaGrange.

The Board of Censors reported favorably on the following applicants: Drs. W. A. J. Anderson, Oxford, Newton county; R. B. Doster, Blakely, Early county; Francis M. Calhoun, Euharlee, Bartow county. These gentlemen were elected members of the Association by adoption of the report.

Dr. S. G. White offered the following:

*Resolved*, That in future this Association will not accept invitations for participation in festivities.

Dr. White explained that such expensive entertainments as the larger cities were in the habit of providing would bear too heavily on the smaller towns where the Association may meet. The matter was discussed by several of the members. Amendments and substitutes were offered by Drs. Love and Bozeman. The whole matter was finally laid on the table.

The President announced as in order the selection of the place for the next annual meeting.

Dr. T. S. Hopkins, of Thomasville, in the name of the citizens of his city, invited the Association to convene next year in that city. He stated that the idea had prevailed that Rome was to be the next place in order, but that Dr. Battey, recognizing the prior claim of Thomasville, had withdrawn in favor of the latter place.

The Association voted unanimously in favor of Thomasville.

The Committee on Publication was called. The Secretary reported that there were four hundred copies of the Transactions published, at a cost of $191 34; that all of the papers referred to the Committee were published, except two articles by Dr. W. A. Love, and one by Dr. J. P. Logan; that these gentlemen had failed, for lack of time or other reason, to place the papers in the hands of the Committee. Report adopted.

The Committee on Medical Education made no report.

The Committee on Medical Literature made no report.

The Committee on Prize Essays, through Dr. J. G. Westmoreland, chairman, reported that no essays had been offered for their consideration.

The Committee for remodeling the Sections of the Congressional Districts submitted the following report:

FIRST DISTRICT.

*Practice of Medicine.*—Drs. A. G. Whitehead, J. M. Johnson.

*Surgery.*—Drs. J. S. McFarland, C. Ganahl, J. B. Reed.

*Gynœcology.*—Drs. J. S. Mims, H. H. Smith, J. P. S. Houston.

SECOND DISTRICT.

*Practice of Medicine.*—Drs. T. S. Hopkins, E. W. Alfriend, J. R. Janes.

*Surgery.*—Drs. R. T. Kendrick, D. S. Brandon, W. L. Davis.

*Gynœcology.*—Drs. B. M. Cromwell, E. P. Ingraham, R. Doster.

THIRD DISTRICT.

*Practice of Medicine.*—Drs. T. F. Walker, J. B. Hinkle, W. A. Greene.

*Surgery.*—Drs. G. F. Cooper, Y. H. Morgan, W. J. Reese.

*Gynœcology.*—Drs. B. M. Cromwell, J. H. Stevens, P. L. Hilsman.

FOURTH DISTRICT.

*Practice of Medicine.*—Drs. A. B. Calhoun, J. A. Urquhart, F. L. Wisdom.

*Surgery.*—Drs. Carlisle Terry, G. J. Grimes, T. F. Brewster.

*Gynœcology.*—Drs. T. J. Word, J. S. Todd, R. B. Ridley.

FIFTH DISTRICT.

*Practice of Medicine.*—Drs. R. L. Roddey, G. M. McDowell, T. J. Mitchell.

*Surgery.*—Drs. G. G. Crawford, K. B. Murchison, Paul Faver.

*Gynœcology.*—Drs. V. H. Taliaferro, W. T. Goldsmith, W. J. Johnson.

SIXTH DISTRICT.

*Practice of Medicine.*—Drs. S. G. White, W. F. Holt, S. H. Gray.

*Surgery.*—Drs. C. B. Nottingham, J. M. Greene, J. B. Hendricks.

*Gynœcology.*—Drs. D. W. Hammond, T. F. Green, C. H. Hall.

SEVENTH DISTRICT.

*Practice of Medicine.*—Drs. W. D. Hoyt, D. H. Payne, J. B. Underwood.

*Surgery.*—Drs. Robert Battey, J. C. Bivings, T. H. P[illegible].

*Gynœcology.*—G. W. Holmes, T. J. Foster, D. G. H[illegible]

EIGHTH DISTRICT.

*Practice of Medicine.*—Drs. W. H. Doughty, A. Mathis, J. A. Ficklin.

*Surgery.*—Drs. DeSassure Ford, L. A. Dugas, J. S. Coleman.

*Gynæcology.*—Drs. H. F. Campbell, M. P. Dedwyler.

NINTH DISTRICT.

*Practice of Medicine.*—Drs. C. W. Long, W. R. Moore, J. W. Bailey.

*Surgery.*—Drs. J. S. Simmons, G. F. Wirsen, B. F. Carlton.

*Gynæcology.*—Drs. E. S. Ray, H. H. Carlton, A. J. Shaffer.

Report adopted.

The following extract from the proceedings of 1872 will show the duties of these committees:

"*Resolved,* That the President of this Association appoint annually, from each Congressional District of this State, a Committee of three on Practice of Medicine, a Committee of three on Surgery, and a Committee of three on Gynæcology, who shall report on some special subject, or subjects, or make reports of cases at their discretion, and present the same at the next meeting of this Association; and that individual members be requested to make voluntary individual reports in like manner. Adopted."

The following resolutions were introduced by Dr. V. H. Taliaferro, and adopted:

1. *Resolved,* That the extraordinary success of the present annual session of this body, not only in the number of attendants and the perfect harmony prevailing, but especially in the very large number of valuable contributions to medical science, is a source of congratulation.

2. *Resolved,* That the thanks of the body be and are hereby tendered to those self-sacrificing conservators of its peace, usefulness and honor—viz, Robert Battey, J. P. Logan, C. B. Nottingham, Juriah Harris and J. G. Thomas—who were so actively instrumental in preserving its integrity at the last session in Columbus.

3. *Resolved,* That the efforts of the Committee of Arrangements in behalf of the medical profession and citizens of Atlanta has largely contributed to the pleasure and profit of the occasion, and will long be held in warm remembrance.

By Dr. D. T. Johnson, of Griffin:

*Resolved,* That the thanks of this Association be and are hereby tendered to the President, Vice-Presidents, Secretary, Treasurer, and Orator, for the discharge of their several duties in such an able and satisfactory manner. Adopted.

The Secretary read, for the Committee on Necrology, biographies of three deceased physicians of Savannah—Drs. P. M. Kollock, E. P. Starr and D. H. Morrison. These papers were prepared and forwarded by Dr. Robert P. Myers, of Savannah. Referred to the Committee on Publication.

Dr. Robert Battey read two papers for Dr. W. H. Philpot, of Talbot county: Case of "Puerperal Convulsions—Attempts at Delivery, and the Cæsarean Section;" case of "Stricture of the Urethra Impermeable to Instruments, Complicated with Perineal Fistula—Operation, Perineal Section." These were referred to the Committee on Publication.

The Association adjourned till 3 o'clock, P. M.

THIRD DAY—AFTERNOON SESSION.

The Association was called to order at 3 o'clock by Dr. E. J. Kirkscey.

Dr. T. F. Brewster recalled two papers which were referred on the previous day without reading, and read them before the body. One paper by himself, "Large Doses Chloral Hydrate Taken with no bad Results;" the other, "Convulsions and Death Produced by Drinking Urine," reported by Thos. S. Mitchell, M.D., Hamilton, Harris county.

Dr. Love, of Atlanta, chairman Committee on Climatology, submitted the caption of an artlcle he was preparing, "A Paper on the Osmotic Action of Medicines Locally Applied in Uterine Diseases, with Results of Treatment in Cases." The paper was referred, after it shall be completed, to the Committee on Publication.

Dr. J. T. Johnson introduced the following—Adopted:

*Resolved,* That all papers intended for publication shall be placed in the hands of the Committee by the first of May.

Dr. J. P. Logan, chairman Committee on Gynæcology of the Seventh District, stated that he had under consideration some papers that he desired to refer to the Committee on Publication, when completed. This privilege was extended him.

By Dr. Wm. Abram Love—Adopted:

*Resolved*, That this Association shall not be considered as endorsing the views of the authors of papers published in connection with the Transactions, unless definitely so endorsed in the proceedings of the Association.

*Resolved*, That the above resolution appear annually upon the title page of our published proceedings.

By Dr. W. T. Goldsmith—Adopted:

*Whereas*, The united action of the members of the profession throughout the State is desirable for the general diffusion of medical information, the cultivation of medical science and the procurement of necessary legislative protection; therefore

*Resolved*, That this body recommend the organization of local societies throughout the State, and invite their annual communication with this body.

*Resolved*, That the Secretary be authorized to forward circulars to prominent medical gentlemen of each county in the State, urging upon them the organization of medical societies in accordance with the objects of the above preamble and resoluions.

Dr. A. W. Griggs offered a resolution thanking Superintendent Mallon, of the Atlanta Public Schools, for the attentions shown; also endorsing the method of classification and teaching, and the dhysical exercises, as demonstrated on the visit of the Association to the Schools. Adopted.

By Dr. W. F. Westmoreland:

*Resolved*, That the clause in the Constitution fixing the time of annual meeting of the Georgia Medical Association be changed to the third Wednesday in April. (To lie over under the rule for one year.)

By Dr. DeSaussure Ford:

*Resolved*, That a special committee of five be appointed to report at the next annual meeting upon the subject of electricity as a remedial agent.

Adopted. Committee: Drs. DeSaussure Ford, R. J. Nunn, T. F. Brewster, W. H. Goodwin and W. M. Charters.

The Committee on Nominations made an additional report, which was adopted:

*Committee of Arrangements*—Drs. T. S. Hopkins, D. S. Brandon, E. P. Taylor.

*Committee of Publication*—Drs. J. T. Johnson, Secretary; W. O'Daniel, Treasurer, Ch. Rauschenberg and J. B. Baird.

*Committee on Prize Essays*—Drs. S. G. White, J. F. Bozeman, G. M. McDowell, W. T. Goldsmith, B. F. Rudisill.

*Committee on Medical Education*—Drs. W. S. Armstrong, E. J. Kirkscey, W. H. Doughty, J. C. C. Blackburn.

*Committee on Climatology and Epidemic Diseases*—Drs. J. A. Urquhart, C. S. Strother, E. W. Alfriend, C. B. Nottingham, R. F. Wright, T. O. Powell, R. P. Myers, J. G. Westmoreland.

*Committee ou State Medical Necrology*—Drs. T. S. Powell, D. W. Hammond, Juriah Harris, Robert Battey.

*Committee on Medical Literature*—Drs. G. G. Crawford, J. R. Boon, R. F. Wright, W. H. Hall, F. A. Stanford.

The following report was submitted by Dr. Bozeman:

"The committee to whom was referred the interesting paper by Dr. Battey, on the subject of the "Removal of the Ovaries," for the causes therein set forth, have not, by reason of limited time afforded to them, been able to give this important subject the thorough and diffusive investigation and discussion to which it is justly entitled. They concur, however, in according to the idea, and its successful execution by its author, the merits of originality, skill and utility. Whether it can be accepted as widely applicable to the relief of suffering from these causes, must yet be confided to the cautious and judicious observation and experiment of the skillful practioner; and they would commend the subject to the critical interest of the profession, and especially to its brilliant author, for additional experiment, and solicit reports from members of this Association on the subject at their next annual meeting.

W. F. Westmoreland,
C. B. Nottingham,
J. F. Bozeman."

By Dr. J. P. Logan—Adopted:

*Resolved*, That the authors of the various papers presented to this body have the privilege of publishing such papers (as may be decided worthy of publication by the committee for that purpose) in any medical journals of the country, crediting the same to this body.

Adjourned to meet at 8 o'clock, for a night session.

THIRD DAY—NIGHT SESSION.

The Association was called to order by the President, Dr. G. W. Holmes.

Dr. J. C. C. Blackburn, of Barnesville, read a paper on the use of oil of winter-green as an anæsthetic. Referred to the Committee on Publication.

The following resolution was adopted:

*Resolved*, That the thanks of the Georgia Medical Association be tendered to Louis Schofield, President of the Rolling Mill, for courtesies extended to the members of the Association.

Br. Dr. E. J. Kirkscey—Adopted:

*Resolved*, That the counties composing the new medical districts be published with the annual proceedings of this body.

The following was introduced and referred to the Board of Censors:

"Dr. E. F. Way, of Hawkinsville, Pulaski county, was expelled from the Georgia Medical Association for irregularities in prac tice; and on being convinced of his violation of the Code, he begs forgiveness and asks to be reïnstated, having abandoned his irregularity, which is vouched for by the subscriber.

*April* 11, 1873. T. F. Walker."

The regular order for the evening, the discussion of cerebrospinal meningitis, was called up.

Dr. J. G. Westmoreland made some remarks sustaining his paper read on the same subject, claiming it to be an acute inflammation, and requiring corresponding active treatment.

He was followed by Drs. S. G. White, E. W. Alfriend, E. D. Alfriend, A. W. Griggs and Wm. O'Daniel. These gentlemen, for the most part, leaned to the "blood poison" theory—that, if an inflammation at all, only of a passive character. Dr. White related several instances pointing to the possibility of its contagiousness.

Dr. W. S. Armstrong stated the appearances observant by himself at many autopsies, mostly made during an epidemic among the soldiers at Mobile during the late war.

Dr. H. V. M. Miller confirmed the observations of Dr. Armstrong; gave an account of an epidemic occurring among the soldiers on the coast during the war. Being in a malarious country, quinine was freely and heroically used, but was alike unsuccessful with the antiphlogistic treatment.

The following resolution by Dr. D. T. Johnson, of Griffin, was adopted:

WHEREAS, We have been the recipient of many and generous hospitalities; therefore,

*Resolved*, The thanks of this Association are due, and are hereby tendered, the citizens and the medical profession of Atlanta, and especially to the Committee of Arrangements, for the many kindnesses shown us during our stay with them, and in leaving, we carry with us a high appreciation and a lasting remembrance for each and all of them.

It was moved and carried that the delegates have power of appointing alternates, in case they were not able to attend.

The President announced the Committee on an Inebriate Asylum—Drs. E. J. Kirkscey, W. T. Goldsmith and S. G. White.

The addresses of the President and Orator were requested for publication.

The Association adjourned to meet in Thomasville, on the first Wednesday in April, 1874.

